# Optimizing a community-engaged multi-level group intervention to reduce substance use: an application of the multiphase optimization strategy

**DOI:** 10.1186/s13063-018-2624-5

**Published:** 2018-04-27

**Authors:** Liliane Cambraia Windsor, Ellen Benoit, Douglas Smith, Rogério M. Pinto, Kari C. Kugler, Newark Community Collaborative Board (NCCB)

**Affiliations:** 1 Newark Community Collaborative Board (NCCB), The University of Illinois at Urbana-Champaign, School of Social Work, 1010 W. Nevada St., Room 2113, Urbana, IL 61801 USA; 2 National Development and Research Institutes, Inc., New York, NY USA; 3 The University of Michigan, School of Social Work, Ann Arbor, MI USA; 4 Department of Biobehavioral Health, Pennsylvania State University, University Park, PA USA

**Keywords:** Critical consciousness theory, Multiphase optimization strategy (MOST), Community based participatory research (CBPR), Substance use disorder (SUD) treatment, Multi-level interventions, Reentry, Factorial design, Formerly incarcerated individuals, Community organizing, health inequalities

## Abstract

**Background:**

Rates of alcohol and illicit drug use (AIDU) are consistently similar across racial groups (Windsor and Negi, J Addict Dis 28:258–68, 2009; Keyes et al. Soc Sci Med 124:132–41, 2015). Yet AIDU has significantly higher consequences for residents in distressed communities with concentrations of African Americans (DCAA - i.e., localities with high rates of poverty and crime) who also have considerably less access to effective treatment of substance use disorders (SUD). This project is optimizing *Community Wise*, an innovative multi-level behavioral-health intervention created in partnership with service providers and residents of distressed communities with histories of SUD and incarceration, to reduce health inequalities related to AIDU.

**Methods:**

Grounded in critical consciousness theory, community-based participatory research principles (CBPR), and the multiphase optimization strategy (MOST), this study employs a 2 × 2 × 2 × 2 factorial design to engineer the most efficient, effective, and scalable version of *Community Wise* that can be delivered for US$250 per person or less. This study is fully powered to detect change in AIDU in a sample of 528 men with a histories of SUD and incarceration, residing in Newark, NJ in the United States. A community collaborative board oversees recruitment using a variety of strategies including indigenous field worker sampling, facility-based sampling, community advertisement through fliers, and street outreach. Participants are randomly assigned to one of 16 conditions that include a combination of the following candidate intervention components: peer or licensed facilitator, group dialogue, personal goal development, and community organizing. All participants receive a core critical-thinking component. Data are collected at baseline plus five post-baseline monthly follow ups. Once the optimized *Community Wise* intervention is identified, it will be evaluated against an existing standard of care in a future randomized clinical trial.

**Discussion:**

This paper describes the protocol of the first ever study using CBPR and MOST to optimize a substance use intervention targeting a marginalized population. Data from this study will culminate in an optimized *Community Wise* manual; enhanced methodological strategies to develop multi-component scalable interventions using MOST and CBPR; and a better understanding of the application of critical consciousness theory to the field of health inequalities related to AIDU.

**Trial registration:**

ClinicalTrials.gov, NCT02951455. Registered on 1 November 2016.

**Electronic supplementary material:**

The online version of this article (10.1186/s13063-018-2624-5) contains supplementary material, which is available to authorized users.

## Background

Rates of alcohol and illicit drug use (AIDU) have been consistently found in the literature to be similar across racial groups [1, 2]. Yet AIDU has significantly greater consequences for residents in distressed communities with concentrations of African Americans (DCAA - i.e., localities with high rates of poverty and crime) in the USA. For instance, these communities suffer both higher incarceration and HIV/hepatitis C virus (HCV) infection rates, particularly among men [3, 4]. Indeed, inequalities in drug-related incarceration rates have devastated DCAAs and impacted African American men more than any other marginalized group [4–7]. Yet residents of DCAAs, in spite of elevated needs, have considerably less access to substance use disorder (SUD) interventions, safe and stable housing, and meaningful employment [4, 8–11]. Most formerly incarcerated people return to distressed communities with high rates of poverty, unemployment, crime, drug trafficking, and depleted social service systems [12]. The vast majority are men; women represent only 6% of the population released from incarceration in Newark, NJ, where this study takes place [12]. These persons have elevated *needs* (e.g., health treatment, housing, employment) and *risks* (e.g., felony labels, weak connections to the labor market but strong connections to illicit markets) [13]. These needs and risks often overwhelm the limited resources available within their families and communities, leading to health inequalities [14–16]. Economic desperation and untreated AIDU problems perpetuate recidivism and elevate crime rates in these neighborhoods [7, 17, 18]. African American men face particularly strong barriers to recovery because they are stereotyped as threats to family and public safety and excluded from employment opportunities that lead to stable housing and financial security [19].

While the etiology underpinning the inequalities discussed above is complex, the cause rests firmly in social determinants of health (e.g., stigma, poverty) [6, 7, 20–22]. Yet, AIDU evidence-based interventions have not paid enough attention to how social determinants of health affect distressed communities differently and often have overlooked marginalized communities’ experiential knowledge and their potential contributions to developing and testing interventions [23–26]. Research suggests that interventions aiming to reduce health inequalities must consider and address social determinants of health [27, 28]. Yet most evidence-based SUD interventions focus solely on changing individual cognition and behavior [25, 29–32].

*Community Wise*, an innovative multi-level, behavioral group intervention, is a significant departure from this model, addressing individual, social and community-level factors simultaneously from a foundation in critical consciousness theory, a well-established framework for mobilizing resistance to social inequalities [33–35]. Interventions that take a comprehensive approach, emphasizing community engagement, can challenge prejudices and strengthen social networks, which in turn can lead to higher levels of employment, housing and financial security, thus benefiting the community as a whole [36–39]. Yet interventions often ignore or minimize environmental factors [23, 40] such as racism, classism, and sexism that have been shown to impact AIDU-related health inequalities and health [7, 41, 42]. New theoretical frameworks targeting the root causes of health inequalities, including stigma and discrimination, are critically needed [43]. Critical consciousness theory not only explains how stigmatization and discrimination impact individual thinking and behavior in general, but also provides a tested strategy to combat the roots of social inequalities [33–35, 44, 45]. However, there are no rigorously tested manuals based on critical consciousness theory available to reduce AIDU among DCAA residents with SUD and a history of incarceration.

Critical consciousness is operationalized as having a deep understanding of how social determinants of health impact AIDU-related health inequalities and using this knowledge to inform critical action that combats health, social, and economic inequalities at the micro level (e.g., cognitive and behavioral processes); the meso level (e.g., relationships with individuals and organizations); and the macro level (e.g., political and cultural processes). We consider that a person who has critical consciousness is empowered to care deeply about both his/her wellbeing and the wellbeing of his/her own communities.

In *Community Wise*, critical consciousness is developed through four main intervention components: (1) core critical-thinking sessions in which participants learn to assess their own thinking about health inequalities and to question assumptions; (2) critical dialogue, where participants attend group meetings and apply critical thinking skills to examine how social determinants of health have impacted their own lives and the health of their communities; (3) development of individual goals through a quality-of-life-wheel exercise; and (4) engagement in capacity-building projects that seek to address social determinants of health. These components of *Community Wise* have potential to increase knowledge about how social determinants of health impact individual behaviors; self-efficacy to engage in change; and actual individual behavior change at the micro level. Moreover, they were designed to strengthen the quality and quantity of positive social relationships at the meso level and to change community norms and structural barriers as communities join together to combat inequalities.

The current study is being conducted by the Newark Community Collaborative Board (NCCB), a network in existence since 2010 that developed and pilot-tested the original *Community Wise* intervention [46, 47]. NCCB members include the principal investigators, co-investigators, service providers, consumers of AIDU, and DCAA residents [48]. Study aims include:

- Aim 1: use a highly efficient experimental design to estimate the unique contribution of candidate experimental components of *Community Wise* in (a) reducing AIDU frequency and (b) increasing the percentage of participants abstinent over 5 months. Candidate components include the type of facilitator delivering the intervention (peer or licensed clinician) and the presence or absence of (a) critical dialogue; (b) quality-of-life-wheel; and (c) capacity building project.
- Aim 2: based on the results from the experiment, make decisions about which components to include in the optimized *Community Wise* intervention that produces the most effective combination of experimental components that can be delivered for less than US$250 per person (as per recommendations by the Substance Abuse and Mental Health Services Administration (SAMHSA) [49].

## Methods/design

Multiphase optimization strategy (MOST) is a new methodological framework that emphasizes efficiency and careful management of resources in the development of multi-component behavioral interventions [50]. Traditional randomized controlled trials (RCT) test an entire intervention’s efficacy against a control group as the first step, which is often followed by more RCTs with large samples per cell to determine which components drive change. However, MOST stresses the optimization of an intervention first, through a process in which various components of the intervention are tested and only the most efficacious combination of experimental component levels are selected, given specific constraints (e.g., sustainable cost) [51–53].

**Fig. 1:**
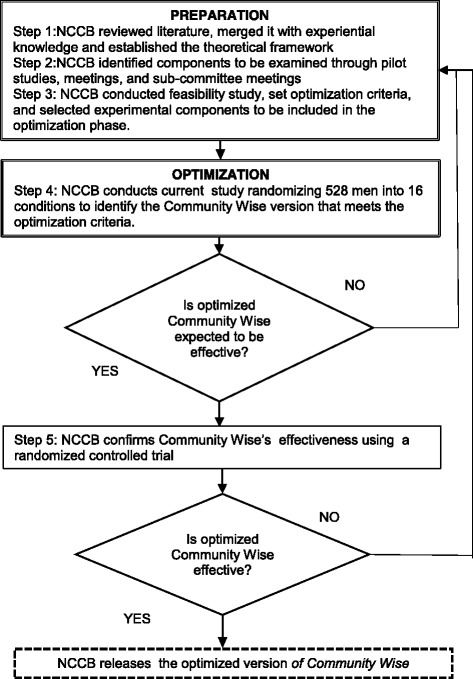
Mapping tool for our application of the multiphase optimization strategy (MOST) under community-based participatory research (CBPR). Adapted from Collins, et al., [69]. NCCB, Newark Community Collaborative Board

Community-based participatory research (CBPR) is an approach to research in which members of the community work as equal partners with academic and/or professional researchers to identify and develop solutions to problems, in order to improve community health and wellbeing [54]. This relationship ensures that research questions and procedures reflect the needs and priorities of the communities themselves, hence facilitating uptake. MOST is an excellent fit with CBPR because MOST gives equal consideration to scientifically rigorous information (e.g. intervention effects) and environmental needs and/or constraints (e.g., sustainability requirements such as cost, dosage, and feasibility). By combining CBPR with MOST, we are (1) including the needs of the community as operationalized by the community; (2) employing rigorous and systematic scientific methods including a factorial design and, subsequently, a rigorous RCT to optimize and test the intervention’s efficacy; and (3) reducing participant burden and use of resources by selecting designs that emphasize efficiency. Figure 1 displays our application of CBPR and MOST in the development and testing of *Community Wise*. This project allows us to complete step 4 in the figure displaying the MOST strategy. Findings will impact the field of public health by developing an optimized multi-level intervention to reduce AIDU that can be adapted to address other health inequalities. Our innovative and rigorous combination of CBPR and MOST will produce action-oriented implications aimed at reducing AIDU and related public health inequalities in DCAAs.

### MOST preparation phase

**Fig. 2:**
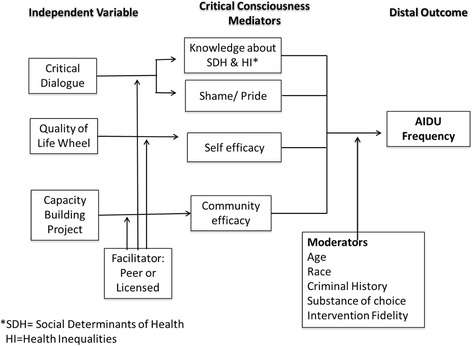
Conceptual model. SDH, social determinants of health; HI, health inequalities; AIDU, alcohol and illicit drug use

The original and complete *Community Wise* intervention was a 12-week, multi-level, community-based, and culturally grounded intervention developed by the NCCB [46]. It was delivered and pilot-tested with 56 participants through weekly two-hour closed group sessions (approximately eight participants per group) [55, 56]. Figure 2 displays the conceptual model developed collaboratively by the NCCB. Once the pilot evaluation was completed, the NCCB members made manual changes and identified the intervention experimental components to be included in the optimization phase. NCCB utilized a systematic process of merging experiential knowledge with data from our pilot evaluation and the scientific literature.

An NCCB taskforce analyzed qualitative and quantitative data from the pilot study to assess feasibility and to identify the intervention experimental components to be included in the optimization phase [55, 56]. Findings informed changes to the manual and the final decision to include a core critical-thinking component and three experimental components: critical dialogue, quality-of-life-wheel, and capacity building projects. These components were selected because findings indicated they seemed to be critical activities for increasing critical consciousness and reducing AIDU among participants [57]; they had a clear financial cost and their individual contribution to AIDU change was unclear. After reviewing the recent literature on the cost of SUD delivery treatment, the NCCB decided to develop a 15-week version of the intervention that can be delivered for less than US$250 per person [49]. Literature on SUD treatment shows that a minimum of 3 months intervention is necessary; yet, the shorter the treatment the better is retention and engagement [40]. These decisions were comparable to strategies that are already being implemented in Newark, NJ and service providers and participants alike agreed that we had a reasonable manual draft. Finally, in the spirit of capacity building and considering the importance of scalability, we are examining if trained peer facilitators (e.g., *Community Wise* graduates) can deliver the intervention with similar results to licensed facilitators. All facilitators receive training in delivering this manualized intervention and participate in ongoing clinical supervision [56].

### MOST optimization phase

**Fig. 3:**
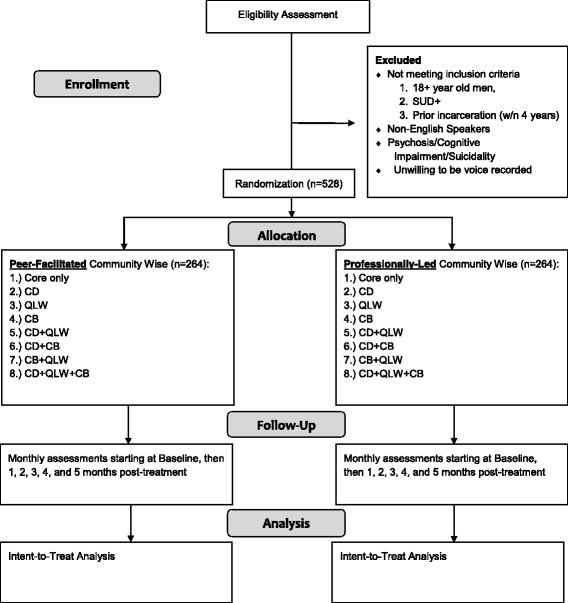
Planned participant flow for the *Community Wise* multiphase optimization strategy (MOST) component screening trial. SUD, substance use disorder; CD, critical dialogue; QLW, quality-of- life-wheel; CB, capacity building project

A 2 × 2 × 2 × 2 factorial experiment is being implemented to evaluate individual and interactive effects of the presence or absence of the following candidate experimental components on AIDU reduction: (a) licensed versus peer facilitator; (b) critical dialogue; (c) quality-of-life-wheel; and (d) capacity building projects. A factorial experiment is being used instead of a classical two-arm randomized controlled trial of the full intervention vs control because the factorial experiment is more efficient in answering the research questions of individual and interaction effects [53]. Figure 3 displays the study design.

### Candidate experimental components

(1) Core component: the core component is delivered through three *Community Wise* group sessions (1, introduction; 2, critical thinking; 3, termination) plus a graduation ceremony. All participants receive this component regardless of experimental condition. The introduction class is essential to set group rules, explain the intervention, and start to create a safe space for participants to engage in dialogue. The facilitator describes the intervention and the group goes over the main concepts (e.g., social determinants of health, critical thinking, community organizing). Participants develop group guidelines and take a pledge to their community to do the work. The critical thinking class is the second session of *Community Wise* and it is essential to helping participants understand critical thinking and knowledge building. Participants learn tools they can use in the group dialogue to challenge pre-conceived ideas, analyze the quality of their own thinking and examine the impact of social determinants of health on health inequalities. For instance, they discuss different types of knowledge, how we build knowledge, and the different value we attribute to different types of knowledge. Participants are encouraged to consider where their beliefs come from, who is affected by their beliefs, and the consequences of their beliefs by engaging in Socratic questioning used by trained intervention facilitators to foster a critical analysis of the community issues raised by participants (e.g., what evidence supports your point of view? If this perspective is true, who benefits and who loses?) [58].

During termination sessions, participants discuss their progress in the program, talk about what they learned and develop plans for the future. The graduation acknowledges participants’ accomplishments, creates a forum where people can learn from community members and experts, and raises awareness in the community, as this is a public event.

The core component sets the foundation for experimental components to work because it creates a safe space and a common language where participants can discuss racism, sexism, classism, and history of marginalization in the context of AIDU and drug traffic, HIV/HCV prevention, and criminal justice. All experimental conditions include the core component.

(2) Critical dialogue refers to group conversations prompted by thematic images that were developed by the NCCB through focus groups [46] in which the facilitators and group members pose critical questions to help participants better understand the impact of social determinants of health on health inequalities. Critical dialogue includes six 2-h weekly sessions. It aims to help participants develop a deeper understanding of how marginalizing processes (e.g., systematic stigma; feelings of rage as victims of discrimination) affect their lives, behavior and communities. Participants randomized to receive this component are encouraged to challenge their pre-conceived ideas and consider different interpretations of the world around them [46]. A great deal of research supports the effectiveness of therapeutic group dialogue in improving myriad health outcomes [59–62] However, critical dialogue was designed to specifically implement critical thinking skills in analyzing the impact of social determinants of health on health outcomes among marginalized groups.

(3) Quality-of-life-wheel refers to a systematic approach to identify long-term personal goals and break them into feasible, measurable, specific steps participants can take on a weekly basis. It was adapted from a tool published in the critical consciousness theory manual and often used by its developer, Paulo Freire, in culture circles [33, 63]. It is also based on SMART goal setting [64, 65]. This component is delivered in the first 60 min of session numbers 4, 6, 8, 10, 12, and 14 (see Table 1). The quality-of-life-wheel aims to increase self-efficacy and help participants develop goals for their future. Participants randomized to receive this component start by completing a circle that is divided into small segments that include a rating scale from 0 to 10. Each pie represents an area of the person’s life (e.g., health, family, education, housing, employment). Participants rate their satisfaction with each area and select the ones they want to focus on. They then learn SMART goal development tools to develop small steps they can implement each week to improve the selected area and reach their goals. They may choose to work on different projects such as improving their relationships with their children, quitting smoking, or paying their debt. During quality-of-life-wheel sessions, participants share their experience implementing their steps with the group and receive feedback and encouragement. Thus, the quality-of-life-wheel can impact AIDU through the use of peer support, problem solving, and goal development.

**Table 1:** Example of *Community Wise* sessions description and grouping into components to be evaluated (for a participant randomized to experimental condition number 1 or number 2)

Session number and theme	Components covered
Session 1: Icebreaker and welcome	Core component
Session 2: Critical thinking class	Core component
Session 3: Solar system	Critical dialogue
Session 4: Empowerment: introduction	Half quality-of-life-wheel and half capacity building project
Session 5: Funhouse mirrors	Critical dialogue
Session 6: Empowerment: implementation	Half quality-of-life-wheel and half capacity building project
Session 7: Walls	Critical Dialogue
Session 8: Empowerment: implementation	Half quality-of-life-wheel and half capacity building project
Session 9: Historical trauma and evaluation	Critical dialogue
Session 10: Empowerment: Implementation & Evaluation	Half quality-of-life-wheel and half capacity building project
Session11: Families/relationships	Critical dialogue
Session 12: Empowerment: implementation and evaluation	Half quality-of-life-wheel and half capacity building project
Session 13: Sexuality	Critical dialogue
Session 14: Empowerment: implementation and evaluation	Half quality-of-life-wheel and half capacity building project
Session 15: Termination and plans for the future	Core component
Graduation ceremony (optional)	Core component

(4) Capacity building projects involve a systematic approach to identify community problems, develop feasible, measurable and specific actions to improve the community, and evaluate their progress. This component is delivered in the second 60 min of session numbers 4, 6, 8, 10, 12, and 14 (see Table 1.) The active component of the capacity building projects draws from community organizing strategies to mobilize communities to foster positive change [66]. It was designed to create collaborative efforts to overcome and dismantle marginalizing processes by building positive social and organizational relationships and community capacity through the development and implementation of community projects aiming to address social determinants of health. Capacity building projects were inspired by one of Freire’s most important strategies in developing critical consciousness: involvement in community projects with community members to improve community conditions and reduce oppression [33]. *Community Wise* participants randomized to receive this component are expected to collaboratively select, design, and implement a feasible project that can address social determinants of health in their community. Many projects can be used for this purpose, including:

- Create a community garden to increase access to healthy foods, foster a walk to raise funds or awareness about health inequalities.
- Conduct a photo-voice project to examine neighborhood housing conditions and educate the public [67, 68].
- Develop a writing group to publish stories of people in the community.
- Organize an event to call attention to or raise funds for a particular community issue.

### Cost estimation

The goal of the optimization phase of MOST is to identify the most promising combination of experimental components in changing an outcome given a set of environmental constraints [69]. In the current study, sustainability was an important consideration and the NCCB wanted to ensure that the intervention would be affordable. Thus, the goal of aim 2 is use the optimization criterion of the most effective intervention that could be delivered for less than US$250 per person. We estimated costs for each experimental component based on projected facilitator salary, participant transportation, space, utilities, and coffee/snacks. Table 2 shows each study condition, the factors being included in each condition, number of intervention sessions, and the estimated total costs of delivering each condition based on the number of sessions it includes.

**Table 2:** Study condition with number of sessions and total cost estimate to deliver each condition

Experimental conditions	CD	QLW	CBP	Facilitator	Sessions (number)	Cost^a^
1	Yes	Yes	Yes	Licensed	15	US$3000
2	Yes	Yes	Yes	Peer	15	US$2235
3	Yes	Yes	No	Licensed	15	US$3000
4	Yes	Yes	No	Peer	15	US$2235
5	Yes	No	Yes	Licensed	15	US$3000
6	Yes	No	Yes	Peer	15	US$2235
7	Yes	No	No	Licensed	9	US$1800
8	Yes	No	No	Peer	9	US$1341
9	No	Yes	Yes	Licensed	9	US$1800
10	No	Yes	Yes	Peer	9	US$1341
11	No	Yes	No	Licensed	15	US$3000
12	No	Yes	No	Peer	15	US$2235
13	No	No	Yes	Licensed	9	US$1800
14	No	No	Yes	Peer	9	US$1341
15	No	No	No	Licensed	3	US$600
16	No	No	No	Peer	3	US$447

*CD* critical dialogue, *QLW* quality-of-life-wheel, *CB* capacity building project

^a^This amount was calculated by adding facilitator cost to the session cost and multiplied by the number of sessions in each component

### Power considerations

Using AIDU frequency as our primary outcome and considering intra-class correlation of 0.60 due to the intervention groups, our sample of 528 participants (with an estimated attrition rate of approximately 10% over the duration of the study) will yield 0.80 power to detect significant differences of *d* = 0.26 main and interaction effects (note that in this type of balanced design, which is analyzed with effect coding, interactions are specified as a regression coefficient and power is the same for main and interaction effects) [70]. We calculated power using a pretest as a repeated measure and an alpha level of 0.05.

NCCB involvement and implementation of CBPR principles:

The NCCB will continue to use a systematic framework [62] in order to sustain the solid CBPR work already in place in Newark, NJ. The NCCB will continue to be governed by its bylaws, hold bi-monthly meetings in a video conferencing equipped room, and conduct business in the form of committees and taskforces. Annual retreats provide a safe space where NCCB members can interact and share their vision for the future of the board, review the bylaws, and address any potential issues. The project budget will be regularly discussed at NCCB meetings to ensure transparency and fairness of resource distribution. NCCB members will be trained as needed to conduct various research-related tasks (from data collection to disseminating findings) throughout the project.

### Research sites

*Community Wise* group meetings and research activities will take place at one of the NCCB’s community-based agency partners who provide substance use and health services to individuals with SUDs. Newark was selected because its residents consistently show poorer health and socio-economic outcomes compared to neighboring areas. The average annual income is US$17,367; 31.9% of residents 25 years of age and older have not completed high school; and 56.2% are African American [71]. In Newark, NJ young adults misuse heroin at twice the national average rates [72]. Over half of the SUD treatment admissions in Essex County are Newark residents [73]. According to the NJ Department of Health and Senior Services, African Americans living in Newark have the highest rate of HIV/AIDS in New Jersey. As of 2011, one in 31 African Americans living in Newark had tested positive for HIV [74].

Study Implementation (Institutional Review Board (IRB), study protocols, and training).

During the first 12 months of the study, the NCCB worked collaboratively to (1) obtain IRB approval for the project; (2) hire, train and certify study staff; (3) refine and finalize assessments; (4) prepare the manuals for each condition; and (5) train facilitators.

### Recruitment and sample selection

The NCCB has established relationships with service agencies in the community and with the population transitioning from incarceration into Newark, NJ. Approximately 10,000 individuals leave prison each year in NJ. Over 95% are men and most live in Newark [12]. The NCCB and project staff post fliers at reentry, SUD, and HIV/HCV service agencies throughout the community and ask individual service providers and *Community Wise* alumni to disseminate information about the study. In addition, research staff encourages potential participants to help distribute the study fliers in their neighborhoods, churches, and other meeting places. Outreach workers approach individuals in key locales in Newark, NJ to spread the word about the study and bring individuals eligible to participate into the agency to complete the clinical screen. Men interested in participating can call the study cell phone number or attend the agency drop-in center. Outreach workers conduct a brief phone screening to obtain self-reported eligibility information including SUD, date of last prison release, age, and contact information.

Eligibility criteria include: over 18 years of age; living in Newark, NJ; having a SUD; agreeing to be audio-recorded during *Community Wise* group sessions; released from incarceration in the past 4 years (timeframe after which the odds of re-incarceration are significantly reduced); [75] English-speaker; and able and willing to provide informed consent. Exclusion criteria include: women; gross cognitive impairment; and severe, unstable mental illness such as untreated psychotic disorders and suicidality.

#### Clinical screen

Following procedures established during the pilot, eligibility is established by an in-person individual interview with a trained master’s-level or doctoral-level clinician using the Mini Mental State Exam [76], the Timeline Follow Back [77], and the Global Appraisal of Individual Needs-Substance Problem Scale (GAIN-SPS) [78]. These instruments are computerized using REDCap software technology [79]. Participants receive US$10 to complete the 40-min clinical screen. Eligible participants are taught by NCCB members about randomization procedures and the different types of treatment they may receive during a study orientation session. Only those who understand and agree to the procedures are consented and invited to complete the baseline assessment.

#### Randomization

Because it is not feasible to offer all 16 experimental conditions simultaneously, the order of implementation of experimental conditions every year of recruitment was randomly selected using a commercially available random number generator [80]. After baseline data collection for a sufficient number of participants to fill multiple groups, a co-investigator with no clinical contact with participants randomizes each participant to one of two groups every time we accumulate at least 22 participant baselines.

#### Assessment

Baseline and follow-up instruments are administered to participants directly via a tablet. All baseline and follow-up data collection occurs in groups of up to 11 participants at a time. The interviewer trains participants in using the computer and the research assistant is available to answer any questions participants may have. This system was successfully used with all participants in our pilot study. Participants receive cash incentives to complete data collection.

#### Primary outcome measures

AIDU are measured during baseline and at 5-monthly follow ups starting 1 month after the first *Community Wise* session is initiated. We use the Timeline Follow Back [77] and a urine toxicology screen (dichotomous) collected by a trained research assistant.

#### Process measures including intervention fidelity, safety, quality assurance, and facilitator competence

REDCap is used to store, manage, monitor and analyze data for component effectiveness, collapsed across all experimental conditions. *Community Wise* group environment and composition are tracked, measured, and compared across groups to assess inter-group and intra-group heterogeneity. *Community Wise* assessments include (1) a checklist completed by facilitators at the end of each *Community Wise* group session to list the activities discussed in each session, and to list each member present and the percent of time that each member attended the session; (2) a use-of-treatment skills measure to track participants’ use of specific tools learned; and (3) measures of participants’ perceptions of helpful experimental components. Costs associated with the delivery of each experimental condition are tracked to obtain data that can inform the development of the optimized *Community Wise*. Participant self-reported data on other services used is obtained through the treatment service review [81]. The NCCB reviews study reports on a bi-monthly basis to address any potential adverse reactions that may occur and any safety concerns that may arise during the sessions, during group supervision, or in the ongoing data analysis. If any participants are found to be unresponsive to the intervention, or getting worse during the course of the study, an NCCB subcommittee discusses the case and the participant’s involvement in the study may be terminated and the participant referred to relevant services in the community.

#### Fidelity

*Community Wise* group sessions are audio recorded so that intervention fidelity can be assessed. Thirty percent of sessions in each group are randomly selected and rated by trained NCCB members on AIDU intervention content, critical consciousness content, facilitator competence, and manual adherence using the fidelity scale that we developed and tested in our pilot work [56]. The principal investigators meet weekly with study staff to review study protocol adherence and address any potential problems. The principal investigators also have weekly supervision meetings with *Community Wise* group facilitators to review results from fidelity ratings and address any potential deviance from the manual.

#### Client satisfaction and feedback

**Fig. 4:**
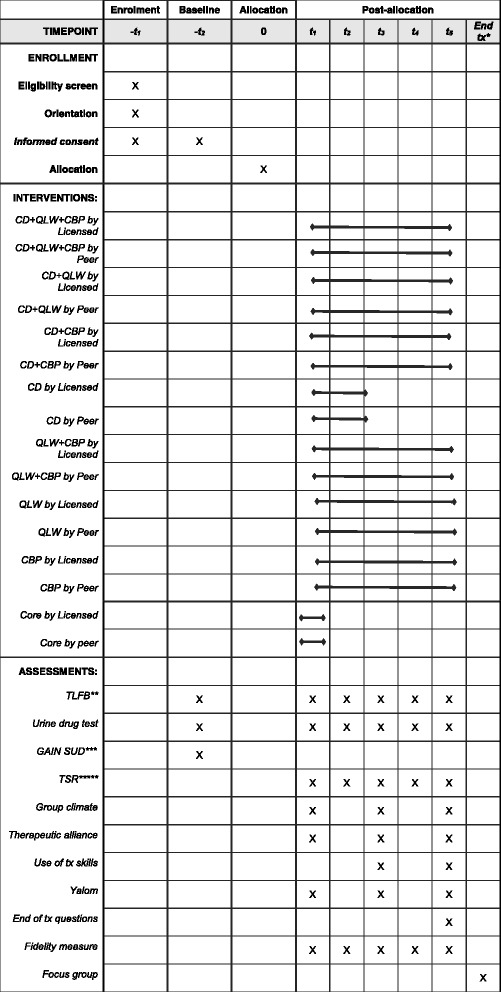
Schedule of enrollment, interventions, and assessments. QLW, quality-of- life-wheel; CBP, capacity building project

We will randomly select 50% of the *Community Wise* groups to participate in 24 focus groups with up to eight participants in each, to gather data on client satisfaction, perceptions about the experimental conditions, and general feedback at the end of each data collection wave. Focus groups are digitally audio-recorded and transcribed. Figure 4 displays the schedule for enrollment, interventions, and assessments.

### Retention

We expect at least 90% retention by the fifth follow up based upon our prior experience with this population [56, 82]. After each assessment, participants are asked to provide extensive locator information, including formal and informal contacts who can reach the participant in a variety of contingencies. The project manager facilitates follow-up interviews for re-incarcerated participants through our collaborations with the NCCB. During the follow-up period, research assistants maintain bi-weekly contact with participants by mail or phone and update locator information. Outreach staff visit community areas known as places frequented by participants to keep in touch and re-engage people in the study.

### Data management and quality control

The principal investigators oversee data management, including data storage, security, random assignment and quality assurance procedures, REDCap, software and hardware, and ensure that all staff adheres to human subjects guidelines. Data management activities and procedures, including data confidentiality, employ the electronic data management systems used in the pilot studies to enhance efficiency, security, and integrity of study data. The NCCB is updated during meetings on data management and adherence to the IRB protocol.

### Data analysis

The NCCB will select a committee of up to three members who will be responsible for working closely with the research team in analyzing the data. NCCB members will play a key role in data analysis and the interpretation of the findings as they bring skills that are unique, different, and complementary to that of academicians [39]. For instance, their experiential knowledge about cultural norms in the Newark community can shed light on the findings. Data analysis results will be presented and reviewed by the full NCCB during board meetings. Qualitative and quantitative findings will be integrated during the decision-making process that will inform the development of the optimized *Community Wise*.

Aim 1 seeks to use an intent-to-treat random sample to examine the effect of each candidate experimental component in reducing AIDU across six time points (baseline and 5-monthly follow ups). Data will first be analyzed descriptively to determine normality, sufficient variability for inferential analyses, missing values, outliers, and illogical values. Erroneous data will be corrected and variable transformations such as taking logarithms or categorization will be used for variables with high skewness, kurtosis, or outliers. We will conduct sensitivity analysis using non-ignorable pattern-mixture and selection models to investigate the robustness of our findings across different models for missing data [83].

For each of the candidate *Community Wise* components we will determine whether there is a difference in AIDU change over time using the baseline as the reference cell. Statistically, these effects will be modeled as (1) component by time interactions, with the fifth follow up as the primary end point and (2) two-way to four-way interactions between components (e.g., critical dialogue by quality-of-life-wheel by capacity building by facilitator type by time interaction). For instance, qualitative data from our pilot study showed that the critical dialogue active component was a promising tool to help participants explore the impact of structural barriers on micro-level and meso-level variables (e.g., AIDU, relationships with others). However, evidence from the literature [84, 85] and from our previous research [56] indicates that changing thinking alone may not be sufficient to change actual behavior. We hypothesize that in addition to changing thinking it is necessary to increase self-efficacy and individual and community capacity to create change. The current study will allow us to test this hypothesis by comparing the individual effect of critical dialogue (designed to change thinking) and interaction effects of critical dialogue with the quality-of-life-wheel (designed to increase self-efficacy) and/or the capacity building project (designed to increase individual and community capacity) active components.

Focus group transcripts will be entered into N-Vivo, a qualitative data processing program. Analysis rooted in phenomenology [86] and grounded theory [87] will include continuous coding, comparison and recoding to yield categories and connect experiences and themes [87]. Analysis will focus on participant reports about their experiences with the experimental conditions, including which components they found most helpful and why and their suggestions for improvements.

Aim 2 seeks to combine NCCB experiential knowledge, findings from the focus groups and results from Aim 1 quantitative analysis to guide decisions about which candidate components to include in the optimized *Community Wise* manual. The NCCB committee will follow MOST’s recommended steps to guide the component selection process [69, 70, 88]. The first step is to examine the components’ main effects that significantly reduce AIDU. Next, these components are considered in light of interaction effects. The next step is to compare these findings with results from the process measures and focus groups. These data will be organized by the data analysis committee and presented at a full NCCB meeting. For instance, if we find that critical dialogue and capacity building projects were the only individual components with a significant effect reducing AIDU, we would consider dropping the quality-of-life-wheel, unless there are qualitative data contradicting this decision. In the second step, if we find that there is a negative interaction effect between critical dialogue and the capacity building project, it means that the combined effect of these two components is less than the sum of its parts.

We will then pose the question: does the component with the smaller effect have a large enough incremental effect to justify its selection? We will address this question by examining the simple effect of the component with lower effect when the component with higher effect is present. We will then discuss these data with the full NCCB in order to decide which components to retain or to drop. This strategy will be used to examine all significant interaction effects. The last step is to consider the cost to deliver the selected components. Our goal is to identify the combination of components that produce the largest effect under US$250 per person. If the combination selected costs more than the allowable amount, the NCCB will examine several scenarios from the second step in which we remove absent/present components to compute the expected outcome based on the regression parameters obtained in the statistical analysis to select the one with the largest effect below US$250. We will explore potential mediation and moderation effects (e.g. treatment adherence, demographics) as feasible, given our study power. This will inform future research to the intervention for specific groups or examine mechanisms of change.

## Discussion

As a community-based participatory research project grounded in critical consciousness theory, this *Community Wise* optimization study leverages experiential knowledge to develop and optimize an intervention to reduce AIDU among a sample of formerly incarcerated men. The use of MOST ensures we are making the most of resources at a time of shrinking state budgets and scarcity of quality services. Further, this is a paradigm shift for building more effective, efficient, and scalable interventions because only those components that are effective and operate within the constraints of cost are included in efficacy trials.

This cutting edge study is operating within the environmental and economic constraints of Newark, NJ, a low-income, majority African American city disproportionately affected by substance use and criminal justice policies. *Community Wise* sessions are led by trained facilitators and designed to foster peer support and to strengthen the capacity of communities to identify and develop their own solutions to health inequalities, including strategies to reform oppressive structures that contribute to such inequalities.

The study was purposefully planned with rapid translation, dissemination, and sustainability in mind. *Community Wise* is envisioned as an efficient and effective model that can ultimately be replicated in the field and scaled-up with high fidelity. Beginning in the research planning stage, and iteratively throughout all phases of the study, we are ensuring that the manual, once determined to be efficacious, can be disseminated among service providers in DCAAs. If it turns out that the peer-facilitated version of the intervention is the most sustainable, efficient, and effective, then *Community Wise* can also lead to employment opportunities for members of the community.

### Challenges and limitations

Due to methodological limitations (e.g., need to focus on a single primary outcome), we are unable to examine change at multiple levels and with multiple outcomes. We focus on AIDU at the individual level because it was a concern identified by the community and it was the most promising outcome in our pilot. Moreover, reduction in health inequalities related to AIDU should follow reduction in AIDU (e.g., HIV risk decreases in the absence of AIDU). Future studies will examine the impact of the optimized *Community Wise* on meso-level and macro-level outcomes. Excluding women was a difficult decision for the NCCB and it is a significant limitation of this study. We will conduct future studies to adapt and test *Community Wise* among women. One of the principal investigators moved away from NJ prior to starting the project. She has been able to maintain close ties to the Newark community and the NCCB by employing computer technologies that allow the team to communicate and collaborate effectively. We plan to recruit men from marginalized populations who may be homeless and who change contact information frequently. The CBPR approach will be critical in maintaining a high level of follow-up retention. Despite these limitations, this study addresses a critical gap in health services research among men with substance use disorders and a history of incarceration residing in DCAAs.

### Future research

The NCCB will design a comprehensive plan to disseminate the study findings to the community at large. Our next study will consist of a randomized, phase III, controlled trial to examine the effectiveness of the optimized *Community Wise* in reducing AIDU compared to a standard of care. Future studies could also include the adaptation and testing of the optimized and effective *Community Wise* manual with other populations and outcomes (e.g., women, adolescents, HIV/HCV prevention/medication adherence, re-incarceration). This can be informed by moderating effects from the current optimization trial to assess the need to develop a different, optimized intervention for different subgroups. Finally, data from this study can be used to adapt the intervention to other communities that may experience different constraints. For instance, additional analysis can be conducted to identify the best component combination that can be delivered at myriad dollar amounts (Additional file 1).

### Trial status

We are currently actively recruiting study participants and collecting data. Participant cluster recruitment and data analysis have not yet begun except for data quality checks and monitoring.

## Additional file

Additional file 1: SPIRIT 2013 checklist: recommended items to address in a clinical trial protocol and related documents. (DOCX 48 kb)

## Abbreviations

AIDU: Alcohol and illicit drug use
CBP: Capacity building projects
CBPR: Community-based participatory research
CD: Critical dialogue
DCAA: Distressed communities with concentrations of African Americans
HVC: Hepatitis C virus
IRB: Institutional Review Board
MOST: Multiphase optimization strategy
NCCB: Newark Community Collaborative Board
QLW: Quality-of-life-wheel
RCT: Randomized controlled trials
